# Pneumonia after Earthquake, Japan, 2011

**DOI:** 10.3201/eid1811.111660

**Published:** 2012-11

**Authors:** Hiroshi Takahashi, Shigeru Fujimura, Satoshi Ubukata, Eizaburo Sato, Makoto Shoji, Mutsuko Utagawa, Toshiaki Kikuchi, Akira Watanabe

**Affiliations:** Saka General Hospital, Tagajo, Japan (H. Takahashi, S. Ubukata, E. Sato, M. Shoji, M. Utagawa);; Tohoku University, Sendai, Japan (S. Fujimura, A. Watanabe),; and Tohoku University School of Medicine, Sendai (T. Kikuchi)

**Keywords:** Tsunami, disaster, earthquake, pneumonia, Haemophilus influenzae, Moraxella catarrhalis, Streptococcus pneumonia, bacteria, Japan

**To the Editor:** The earthquake that occurred in Japan on March 11, 2011, triggered an extremely destructive tsunami (*1*), which destroyed cities along the Pacific coastline in the Tohoku area and resulted in the loss of >19,000 human lives. Water from the tsunami inundated ≈33.7% of Tagajo City (population ≈61,000) and caused 188 deaths. Many local residents were left without lifeline utilities, including electricity, gas, water, or any means of transportation and thus were forced to live in crowded shelters or limited small spaces (e.g., the upper floor of their home); ≈11,000 persons were displaced from their damaged or destroyed homes to crowded school gymnasiums or community centers. In March, the mean daily maximum air temperature in Tagajo City was cold (8°C/46.4°F). After the earthquake, cases of pneumonia increased rapidly.

Saka General Hospital is located in this region near the coast. The destruction around the hospital was so severe that persons were without electricity, water, gas, and fuel for several weeks. Fortunately, the hospital laboratory was almost completely functional and could perform bacterial and other tests at a near-normal level, despite the earthquake. However, several other hospitals in the area were severely damaged and thus had difficulty treating patients with severe pneumonia.

To determine the characteristics of pneumonia after the earthquake, we conducted a retrospective study of patients who had pneumonia during the 6 weeks before the earthquake and the first 9 weeks after the earthquake. To identify patients with pneumonia, we checked all chest radiographs and computed tomography scans of adult patients (>16 years of age) who had visited the hospital. We examined clinical and bacteriologic data for these patients. We excluded from the study patients without sputum culture and patients with other conditions, such as lung cancer, pulmonary infarction, or cardiac failure.

During the 6 weeks before the earthquake, pneumonia had been diagnosed for 49 adults (controls), and within the 9 weeks after the earthquake, community-acquired or health care–associated pneumonia was newly diagnosed for 172 adults. Patient data from 2 pre-earthquake periods and 3 postearthquake periods are shown in the Table. Although the number of patients with pneumonia in the first 3 weeks after the earthquake increased sharply, no substantial differences were noted in mean age, death rates, or underlying concurrent conditions among these patients. The interval between the onset of respiratory signs and symptoms and a diagnosis of pneumonia did not increase after the earthquake. The proportion of patients who received antimicrobial drugs before the diagnosis of pneumonia (premedication) in the early postearthquake period did not differ significantly. The number of patients with pneumonia peaked in the first 3 weeks after the earthquake, followed by a gradual decrease starting from 4 weeks after the earthquake.

**Table:** Characteristics of patients with pneumonia before and after the earthquake and tsunami, Japan, 2011*

Characteristic	Weeks before disaster			Weeks after disaster		
	4–6	1–3		1–3	4–6	7–9
Patients with pneumonia, no.	26	23		83	51	38
CAP	20	19		57	39	24
HCAP	6	4		26	12	14
Isolates from sputum culture, no. (%)						
* Streptococcus pneumoniae*	8 (30.8)	2 (8.7)		19 (22.9)	10 (19.6)	4 (10.5)
* Haemophilus influenzae*	4 (15.4)	4 (17.4)		27 (32.5)	8 (15.7)	4 (10.5)
* Moraxella catarrhalis*	1 (3.8)	0		26 (31.3)	9 (17.7)	3 (7.9)
Purulent sputum, (Geckler 4 or 5), %	14 (53.8)	10 (43.5)		51 (61.4)	24 (47.1)	16 (42.1)
Mean age, y	73.7	76.0		75.5	76.0	74.9
Location of patient at illness onset, no.						
Shelter	NA	NA		36	13	6
Own or friend’s home	NA	NA		37	29	23
Nursing home / home visit by doctor	NA	NA		10	9	9
Patient’s hospital status, no.						
New patient	7	3		32	19	20
Routinely examined at hospital	19	20		51	32	18
Rate of hospital admission, %	76.9	73.9		77.1	64.7	78.9
Deaths, no. (%)	3 (11.5)	1 (4.3)		6 (7.2)	2 (3.9)	3 (7.9)
Underlying disease, %						
Respiratory disease	8(30.8)	12 (52.2)		29 (34.9)	19 (37.3)	13 (34.2)
Other	18 (69.2)	11 (47.8)		54 (65.0)	31 (60.8)	23 (60.5)
Healthy	1 (3.8)	0		8 (9.6)	5 (9.8)	2 (5.3)
Interval from onset to diagnosis, mean no. days	3.96	2.43		2.51	3.22	2.89
Antimicrobial premedication, no., %	2 (7.7)	0		4 (4.8)	11 (21.6)	3 (7.9)

*CAP, community-acquired pneumonia; HCAP; health care–acquired pneumonia; NA, not applicable.

Chest radiographs were taken and hematologic examinations were performed for all patients; computed tomography of the chest and rapid diagnostic tests for influenza were performed for 42.2% and 54.2% of 83 patients, respectively, who had pneumonia in the early postearthquake period. During the first 3 weeks after the earthquake, *Haemophilus influenzae* and *Moraxella catarrhalis* were more predominant than *Streptococcus pneumoniae*; most strains were isolated from purulent sputum specimens. In contrast, pneumonia caused by enterobacteria, staphylococci, or atypical pathogens did not increase after earthquake.

Detection rates of *H. influenzae* remained constant at 15.4% (4/26 patients); before the earthquake the rate was 17.4% (4/23), and soon after the earthquake it increased to 32.5% (27/83). Detection rates of *M. catarrhalis* increased from 0–3.8% before the earthquake to 31.3% (26/83) after the earthquake (p<0.01). These bacterial strains were isolated widely from refugees at shelters and from persons living at home without running water and/or electricity. Soon after the earthquake, it was thought that infections with these strains were not part of a localized outbreak but were widespread in the region. Most patients from whom *M. catarrhalis* was isolated were located throughout the area flooded by the tsunami. In contrast, many patients with *H. influenzae* were mainly located outside the flooded area. There was no regional imbalance in isolation of *S. pneumoniae*.

It was reported that living in a multiple-bedroom residence and the winter season were risk factors for outbreaks of *M. catarrhalis* (*2*–*4*). Similar outbreaks of *H. influenzae* infections were reported (*4*,*5*). Cold shock at a physiologically relevant temperature of 26°C promotes *M. catarrhalis* adherence to upper respiratory tract cells and can contribute to virulence (*6*).

The possibility of a pseudo-epidemic must also be considered. The substantial increase in the number of new patients at Saka General Hospital, as a result of the severe damage to other hospitals in this area and the changed patient profiles (community-acquired pneumonia, hypothermia, trauma), might have largely affected the etiology of pneumonia. We found no increase in cases of severe pneumonia caused by resistant bacteria or aspiration pneumonia in elderly patients. We conclude that multiple localized small community outbreaks might have occurred widely in this area after earthquake.
